# Infectious Bronchitis Virus Generates Spherules from Zippered Endoplasmic Reticulum Membranes

**DOI:** 10.1128/mBio.00801-13

**Published:** 2013-10-22

**Authors:** Helena J. Maier, Philippa C. Hawes, Eleanor M. Cottam, Judith Mantell, Paul Verkade, Paul Monaghan, Tom Wileman, Paul Britton

**Affiliations:** The Pirbright Institute, Compton Laboratory, Compton, United Kingdom^a^; The Pirbright Institute, Pirbright Laboratory, Pirbright, United Kingdom^b^; Biomedical Research Centre, Faculty of Health, School of Medicine, University of East Anglia, Norwich, United Kingdom^c^; Wolfson Bioimaging Facility, School of Biochemistry and Physiology & Pharmacology, University of Bristol, Bristol, United Kingdom^d^

## Abstract

Replication of positive-sense RNA viruses is associated with the rearrangement of cellular membranes. Previous work on the infection of tissue culture cell lines with the betacoronaviruses mouse hepatitis virus and severe acute respiratory syndrome coronavirus (SARS-CoV) showed that they generate double-membrane vesicles (DMVs) and convoluted membranes as part of a reticular membrane network. Here we describe a detailed study of the membrane rearrangements induced by the avian gammacoronavirus infectious bronchitis virus (IBV) in a mammalian cell line but also in primary avian cells and in epithelial cells of *ex vivo* tracheal organ cultures. In all cell types, structures novel to IBV infection were identified that we have termed zippered endoplasmic reticulum (ER) and spherules. Zippered ER lacked luminal space, suggesting zippering of ER cisternae, while spherules appeared as uniform invaginations of zippered ER. Electron tomography showed that IBV-induced spherules are tethered to the zippered ER and that there is a channel connecting the interior of the spherule with the cytoplasm, a feature thought to be necessary for sites of RNA synthesis but not seen previously for membrane rearrangements induced by coronaviruses. We also identified DMVs in IBV-infected cells that were observed as single individual DMVs or were connected to the ER via their outer membrane but not to the zippered ER. Interestingly, IBV-induced spherules strongly resemble confirmed sites of RNA synthesis for alphaviruses, nodaviruses, and bromoviruses, which may indicate similar strategies of IBV and these diverse viruses for the assembly of RNA replication complexes.

**IMPORTANCE ** All positive-sense single-stranded RNA viruses induce rearranged cellular membranes, providing a platform for viral replication complex assembly and protecting viral RNA from cellular defenses. We have studied the membrane rearrangements induced by an important poultry pathogen, the gammacoronavirus infectious bronchitis virus (IBV). Previous work studying closely related betacoronaviruses identified double-membrane vesicles (DMVs) and convoluted membranes (CMs) derived from the endoplasmic reticulum (ER) in infected cells. However, the role of DMVs and CMs in viral RNA synthesis remains unclear because these sealed vesicles lack a means of delivering viral RNA to the cytoplasm. Here, we characterized structures novel to IBV infection: zippered ER and small vesicles tethered to the zippered ER termed spherules. Significantly, spherules contain a channel connecting their interior to the cytoplasm and strongly resemble confirmed sites of RNA synthesis for other positive-sense RNA viruses, making them ideal candidates for the site of IBV RNA synthesis.

## IMPORTANCE

All positive-sense single-stranded RNA viruses induce rearranged cellular membranes, providing a platform for viral replication complex assembly and protecting viral RNA from cellular defenses. We have studied the membrane rearrangements induced by an important poultry pathogen, the gammacoronavirus infectious bronchitis virus (IBV). Previous work studying closely related betacoronaviruses identified double-membrane vesicles (DMVs) and convoluted membranes (CMs) derived from the endoplasmic reticulum (ER) in infected cells. However, the role of DMVs and CMs in viral RNA synthesis remains unclear because these sealed vesicles lack a means of delivering viral RNA to the cytoplasm. Here, we characterized structures novel to IBV infection: zippered ER and small vesicles tethered to the zippered ER termed spherules. Significantly, spherules contain a channel connecting their interior to the cytoplasm and strongly resemble confirmed sites of RNA synthesis for other positive-sense RNA viruses, making them ideal candidates for the site of IBV RNA synthesis.

## Introduction

Viruses rely on numerous aspects of the host cell machinery to support each stage of their replication cycle. In the case of positive-sense single-stranded RNA viruses, expression of viral proteins results in the characteristic restructuring of cellular membranes required for the assembly of viral RNA synthesis machinery. Assembly of viral replication complexes or virus factories on cellular membranes is thought to increase efficiency of RNA synthesis by concentrating viral enzymes at specific sites. The precise mechanism of membrane rearrangements for many viruses is poorly defined, but understanding this crucial step in virus replication could have important implications for designing next-generation vaccines and antiviral therapeutics.

The types of membrane rearrangements induced by positive-sense RNA viruses differ between virus families (reviewed in references 1 to 4). Membrane networks fall into two main categories termed double-membrane vesicles (DMVs), which are sealed from the cytoplasm, and invaginated vesicles or spherules, which have a channel connecting to the cytoplasm (3). Viruses inducing DMVs include hepatitis C virus (5, 6) and poliovirus (7, 8), whereas Brome mosaic virus (BMV), flock house virus (FHV), Semliki Forest virus (SFV), and some flaviviruses induce spherules or invaginated vesicles (9–15).

Studies of the membrane rearrangements induced during replication of three betacoronaviruses, mouse hepatitis virus (MHV), severe acute respiratory syndrome coronavirus (SARS-CoV), and the recently identified Middle East respiratory syndrome coronavirus (MERS-CoV) (16–18), have identified DMVs (18–24), as well as reticular inclusions (25) or convoluted membranes (18, 23, 24). However, the location of viral replication complexes and viral RNA synthesis remains unclear. Immunoelectron microscopy (EM) has shown that double-stranded RNA (dsRNA) is localized within the interior of the DMVs (23); however, electron tomography (ET) of DMVs generated by SARS-CoV failed to detect any connections to the cytoplasm that would allow transport of nascent RNA from the DMVs to the cytoplasm to reach the sites of translation or virus assembly. Instead, DMVs are connected to one another and are connected to convoluted membranes and the ER via their outer membranes (23). In addition, viral replicase proteins have been found to be concentrated outside the DMVs as well as on the convoluted membranes, although some signal could be detected inside DMVs in some studies (19–24).

Infectious bronchitis virus (IBV) is a gammacoronavirus that infects poultry, causing large economic losses to global poultry industries as a result of poor meat quality and poor egg production and egg quality. To date, little work has been performed to determine the onset of different processes during the IBV replication cycle. Furthermore, although a study was carried out in the 1970s to characterize modifications to the host cell during IBV replication (26), to our knowledge, no further studies have been performed to identify in detail the rearrangement of cellular membranes by IBV. Here we report a description of the course of viral RNA and protein synthesis and particle release, together with a detailed analysis of cellular structures and membrane rearrangements induced by IBV, with a particular focus on identifying potential sites of virus RNA synthesis that would allow the transfer of nascent viral mRNA and genome to sites of translation and packaging. Significantly, this work was performed not only in a continuous cell line, similar to previous work with other coronaviruses, but also in primary avian cells and in epithelial cells of the chicken trachea using *ex vivo* organ cultures. Interestingly, we identified in all three cell types membrane rearrangements that appear to be novel to infection by IBV, regions of zippered ER, and associated spherules. ET identified that the spherules are connected to the zippered ER and, significantly, contain a channel connecting their interior to the cell cytoplasm. DMVs were also observed in IBV-infected cells, and ET confirmed that a small proportion of the IBV-induced DMVs are connected to the ER via their outer membranes, although many were observed with no membrane connections.

## RESULTS

### Characterization of virus replication.

The profile of IBV RNA synthesis was determined using a 2-step quantitative reverse transcription-PCR (qRT-PCR) assay. Chick kidney (CK) cells were infected with IBV at a multiplicity of infection (MOI) of 10, and total RNA was harvested at hourly intervals. Quantitative RT-PCR was performed using primers specific for N protein subgenomic RNA (sgRNA), as a determinant for the level of subgenomic RNA, and for the 5′ untranslated region (UTR) of genomic RNA (gRNA), as a determinant for the level of full-length IBV RNA transcripts. Absolute quantitation was performed using a plasmid containing the region of interest to provide a known copy number standard curve, and data were expressed as relative RNA copy numbers based on cDNA input. Results showed that IBV-derived RNA was detectable as early as 2 h postinfection (hpi) (Fig. Ai and Aii). The level of IBV genomic RNA increased very little from 2 to 4 hpi but then increased from 4 to 7 hpi. Genomic RNA began to reach a steady state by 8 hpi (Fig. Ai). Analysis of N protein sgRNA showed that the level of this RNA increased steadily from 2 to 7 hpi. The N sgRNA also began to reach a steady state by 8 hpi (Fig. Aii). Overall, we observed that IBV sgRNA levels began to increase early in infection (2 to 7 hpi) whereas genomic RNA levels increased later in infection (4 to 7 hpi).

**FIG 1:**
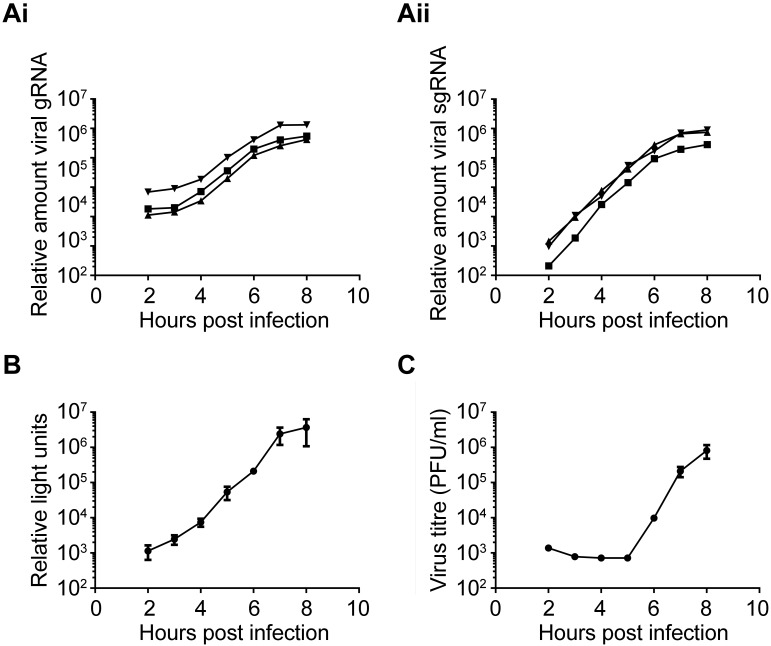
Characterization of virus replication. (A) CK cells were infected with IBV, and total RNA was extracted at hourly intervals. Levels of genomic RNA (gRNA; Ai) and N sgRNA (Aii) were measured by 2-step qRT-PCR. Data from three independent experiments are shown. (B) CK cells were infected with recombinant IBV BeauR-hrLuc-Δ5a expressing Renilla luciferase in place of gene 5a. Protein lysates were harvested at hourly intervals, and Renilla luciferase activity was measured. Data representing the means and standard deviations of the results of three independent experiments are shown. (C) CK cells were infected with IBV and cell supernatants harvested at hourly intervals. Release of progeny virus was determined by a plaque assay. Data representing the means and standard deviations of the results of three independent experiments are shown.

In order to determine concomitant IBV protein synthesis, we utilized a recombinant IBV, BeauR-hRluc-Δ5a, in which the 5a accessory gene had been replaced by the Renilla luciferase reporter gene (27). CK cells were infected with BeauR-hRluc-Δ5a, and protein samples were harvested at hourly intervals for determination of Renilla luciferase activity. Luciferase activity could be detected from 2 hpi, and luciferase activity increased steadily over the 8-h period analyzed, reaching steady-state activity between 7 and 8 hpi (Fig. 1B).

Finally, to determine the onset of progeny virus release from IBV-infected cells, primary CK cells were infected with IBV and cell supernatants were harvested at hourly time points. The presence of infectious progeny virus released from infected cells was assayed by a plaque assay. IBV progeny virus was detected in CK cell supernatants from 6 hpi, and the amounts of progeny virus detected continued to increase through 8 hpi (Fig. 1C).

### A minor population of viral polymerase colocalizes with dsRNA.

**FIG 2:**
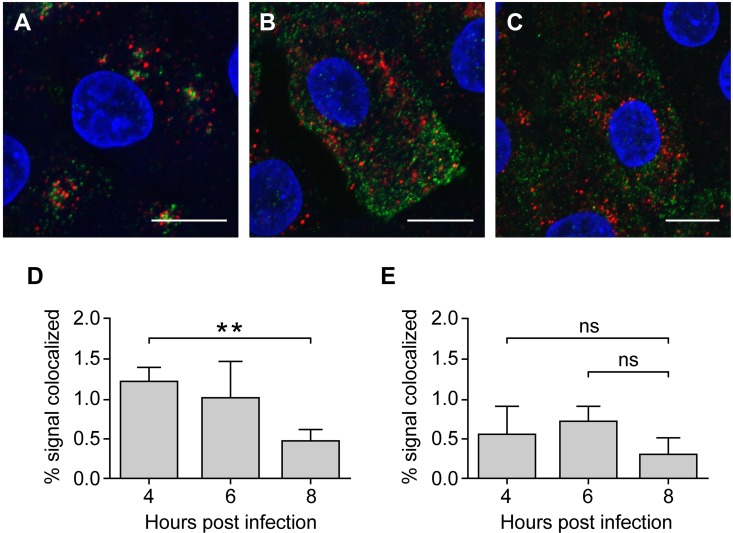
Minor populations of dsRNA and Nsp12 colocalize. (A, B, and C) CK cells were infected with IBV and fixed using 4% paraformaldehyde at 4 (A), 6 (B), or 8 (C) hpi. Cells were labeled with anti-dsRNA (red) and anti-nsp12 (green). Nuclei were stained with DAPI (blue). Bars indicate 10 µm. (D and E) Colocalization of dsRNA (D) and nsp12 (E) was quantified using the Imaris Coloc module. Means and standard deviations of the results of three independent experiments are shown. Ns, not significant; **, *P* ≤ 0.01 (Student’s *t* test).

The presence of dsRNA has previously been used as a marker for nidovirus replication complexes and has been shown in SARS-CoV- and equine arterivirus (EAV)-infected Vero cells to localize predominantly to the inside of DMVs (23, 28). However, the role of dsRNA as a marker for sites of replication of nidoviruses remains unclear. While dsRNA was found to colocalize with newly synthesized RNA at early time points following MHV infection, at later time points, colocalization reduced, and certainly some sites of dsRNA accumulation may represent storage of nonfunctional RNA (29). In addition, as no pore or channel has been identified connecting the interior of DMVs with the cytoplasm (23, 28), it is unclear how newly synthesized RNA exits DMVs to be transported to sites of translation or virus packaging. As an alternative means of identifying sites of IBV replication, we compared the locations of IBV RNA-dependent RNA polymerase-associated nonstructural protein 12 (Nsp12) and dsRNA. Both dsRNA and Nsp12 could be detected at 4 hpi in a small number of defined foci in the cytoplasm (Fig. 2A), and at 6 and 8 hpi, the numbers of both dsRNA and Nsp12 foci increased and the foci spread throughout the cytoplasm (Fig. 2B and C). However, colocalization analysis performed using the Imaris software Coloc module showed that less than 1.5% of the dsRNA and Nsp12 signal was colocalized at any time point, with a Pearson’s coefficient at 4 hpi of 0.77 (±0.24), at 6 hpi of 0.61 (±0.12), and at 8 hpi of 0.69 (±0.14) (Fig. 2D and E). In addition, while the percentage of Nsp12 that colocalized with dsRNA remained constant over the course of infection (Fig. 2E), the overall percentage of colocalized dsRNA signal decreased (Fig. 2D). Therefore, only a minor population of IBV polymerase, Nsp12, and IBV-induced dsRNA colocalized throughout infection.

### IBV induces zippered ER and spherules in infected cells.

**FIG 3:**
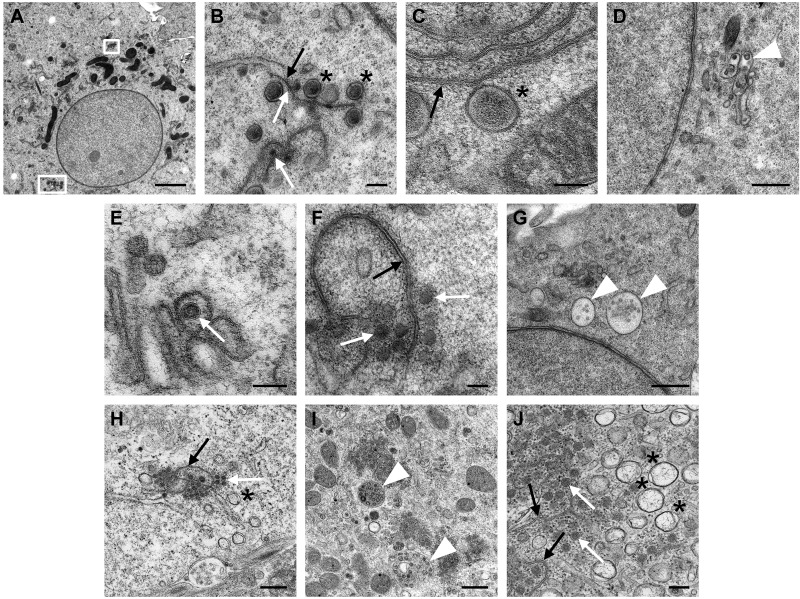
Zippered ER, spherules, and double-membrane vesicles are induced by IBV in CK cells. CK cells were infected with IBV, and at 16 hpi (A to I) or 24 hpi (J), cells were fixed by high-pressure freezing (A to G) or chemical fixation (H and J). Double-membrane vesicles are marked with asterisks, spherules are marked with white arrows, zippered ER is marked with black arrows, and virus particles in vesicles are marked with white arrowheads. The white boxes in panel A highlight regions of IBV-induced membrane rearrangements. The region marked by the lower box is expanded in panel B. Scale bars: A, 2 µm; B and J, 200 nm; C, E, and F, 100 nm; D and G to I, 500 nm.

To observe the precise nature of the membrane rearrangements induced by IBV in avian cells, primary CK cells were infected with IBV and fixed by either high-pressure freezing (HPF) or chemical fixation at 16 or 24 hpi and analyzed by transmission electron microscopy (TEM). IBV induced a number of different membrane rearrangements during infection (Fig. 3). The most striking observation was the presence of zippered membranes (Fig. 3A to C, E, F, H, and J) generated as a result of the zippering together of the ER cisternae (Fig. 3B and H). In addition, small double-membrane spherule-like structures were observed to be associated with the zippered ER (Fig. 3B, E, F, H, and J). Regions surrounding the zippered ER appeared to be more electron dense than the cytoplasm, indicating the accumulation of protein in these areas. Analysis of the spherules showed they had an average diameter of 80.3 nm (±9.2 nm) in chemically fixed samples and of 62.3 nm (±12.0 nm) in high-pressure frozen samples and consisted of a double membrane using both fixation methods (Fig. 3B, E, F, H, and J). Despite being of a size comparable to that of virus particles (Fig. 3D, G, and I), spherules had clear differences in morphology and were, therefore, not intermediates in virus particle formation. In addition to these novel structures, DMVs, previously observed in cells infected with other coronaviruses (19–21, 23, 24) and with an arterivirus, EAV (28, 30, 31), were also observed in IBV-infected cells, confirming that a gammacoronavirus as well as betacoronaviruses induces the formation of DMVs. DMVs were identified in IBV-infected cells fixed by either high-pressure freezing (Fig. 3B and C) or chemical fixation (Fig. 3H and J), in which the appearances of these structures differed. In chemically fixed cells, the IBV-induced DMVs resembled those seen previously for other coronaviruses, with lacy contents and the two membranes separated from one another in places. The average diameter of the IBV-induced DMVs observed in the chemically fixed cells was 222.6 nm (±24.1 nm). In cells fixed by high-pressure freezing, the average diameter of the DMVs was 179.0 nm (±26.8 nm), and the content was well preserved and was observed as an electron-dense core and not simply as engulfed cytoplasm. This is similar to the DMVs observed in EAV-infected cells that had been fixed by high-pressure freezing (28). Furthermore, fixation by high-pressure freezing revealed that the membranes of the IBV-induced DMVs were tightly apposed and remained in contact with one another. Analysis of the IBV-infected cells, using both methods of fixation, did not identify any association of the outer membranes of the IBV-induced DMVs, or any other membrane rearrangement, with ribosomes (Fig. 3B, C, E, F, H, and J). Both fixation methods showed that the spherules had an appearance distinct that of DMVs with no electron-dense core, and no intermediate form was observed, suggesting that spherules are not progenitors in DMV formation. Furthermore, spherules were never observed to be associated with membranes other than the zippered ER, including nonzippered regions of ER. Progeny IBV virus could be seen budding into vesicles, and virus particles were observed to be present inside vesicles, either as single particles or as numerous particles per vesicle (Fig. 3D, G, and I). The zippered ER and spherules observed in IBV-infected cells appear to be novel and distinct from the convoluted membranes observed in cells infected with SARS-CoV, MHV, or MERS-CoV. We did not observe any membrane rearrangements in IBV-infected CK cells, analyzed using either fixation method, which resembled the convoluted membranes observed in cells infected with the three betacoronaviruses.

**FIG 4:**
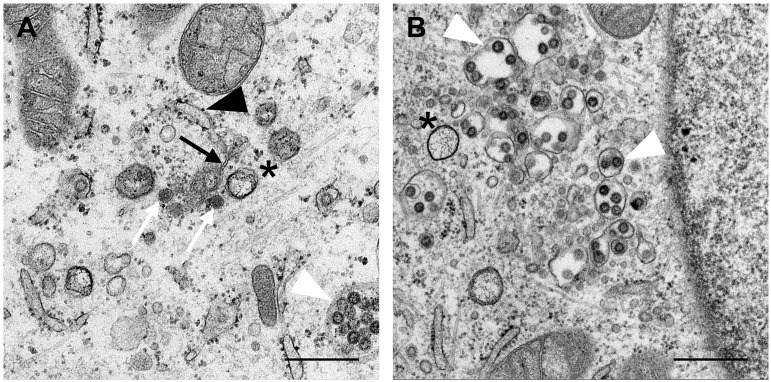
Zippered ER, spherules, and double-membrane vesicles are induced by IBV in Vero cells. Vero cells were infected with IBV, and at 16 hpi, cells were chemically fixed. Double-membrane vesicles are marked with asterisks, spherules are marked with white arrows, zippered ER is marked with black arrows, virus particles in vesicles are marked with white arrowheads, and ribosomes are marked with black arrowheads. Scale bars indicate 100 nm (A) or 200 nm (B).

Previous work studying membrane rearrangements induced during coronavirus infection has used well-characterized continuous cell lines, rather than primary cells (18–21, 23, 24). Therefore, to determine whether the differences in the membrane structures observed in IBV-infected cells compared to previous observations in SARS-CoV-, MHV-, or MERS-CoV-infected cells occurred as a result of the cells used, experiments were performed in mammalian Vero cells. Cells were infected with IBV, and at 24 hpi the cells were chemically fixed and the presence of membrane rearrangements was investigated by TEM. Analysis of the IBV-infected Vero cells identified IBV-induced structures similar to those observed in CK cells (Fig. 4A) as well as virus particles in vesicles (Fig. 4B). In addition, although ribosomes could be detected in association with ER membranes, no ribosomes were found in association with IBV-induced membrane rearrangements (Fig. 4A). Overall, our results demonstrated that IBV induces novel membrane rearrangements, regions of zippered ER, and small double-membrane spherules as well as DMVs in both avian primary and mammalian cell lines. The novel structures, to our knowledge, have not been previously observed in cells infected with three other coronaviruses.

### Zippered ER and spherules are detected at 7 hpi.

**FIG 5:**
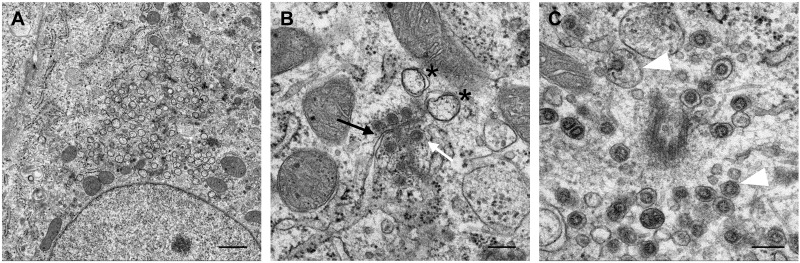
IBV-induced membrane rearrangements can be detected at 7 hpi. CK cells were infected with IBV and chemically fixed at hourly intervals. Double-membrane vesicles (*), spherules (white arrow), and zippered ER (black arrow), as well as virus particles in vesicles (white arrowhead), were detectable at 7 hpi. Scale bars indicate 1 µm (A) or 200 nm (B and C).

At 7 hpi, there was optimum production of sgRNA and gRNA, a good signal for dsRNA and Nsp12 detection by immunofluorescence, and viruses were emerging from infected cells. Therefore, we chose 7 hpi to confirm the presence of membrane rearrangements at this peak time point of virus infection and to capture all events in replication, assembly, and release. Primary CK cells were infected with IBV and at 7 hpi were chemically fixed and analyzed by TEM. IBV-induced membrane rearrangements were observed in a large perinuclear area (Fig. 5A), with zippered ER, spherules, and DMVs all detectable (Fig. 5B). Virus particles were also detected inside vesicles (Fig. 5C). Additional samples were prepared at earlier time points postinfection (1 to 6 hpi; data not shown), but despite searching 200-cell sections, no rearranged membranes or virus particles could be detected at earlier time points. The difficulty in detecting rearranged membranes and progeny virus by TEM is most likely due their low occurrence earlier in infection.

### Zippered ER and spherules observed in IBV-infected *ex vivo* tracheal organ cultures.

**FIG 6:**
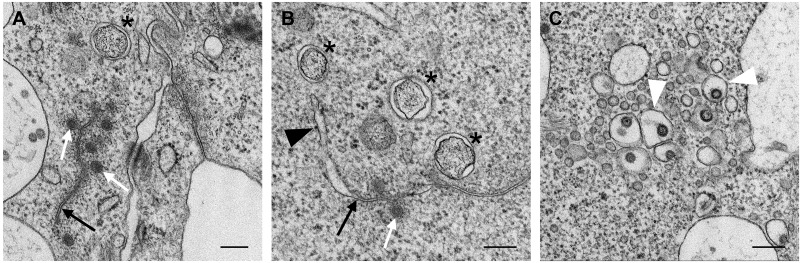
IBV infection induced zippered ER, spherules, and double-membrane vesicles in *ex vivo* tracheal organ culture (TOC). TOCs were infected with IBV and at 24 hpi were chemically fixed. Double-membrane vesicles (*), spherules (white arrow), and zippered ER (black arrow) (A and B), as well as virus particles in vesicles (white arrowhead) (C), could be detected. Ribosomes could be detected on ER membranes (B, black arrowhead). Scale bars indicate 200 nm.

Although primary avian cells were used in this work to identify IBV-induced membrane structures, IBV primarily infects the epithelial cells lining the respiratory tract of chickens. Therefore, to determine whether similar structures are also present in cells of the avian airway, tracheal organ cultures (TOCs) were infected with IBV and at 16 hpi were chemically fixed and analyzed by TEM. Consistent with observations from primary CK cells, IBV infection of tracheal epithelial cells induced zippered ER and double-membrane spherules associated with the zippered ER, as well as DMVs and virus particles (Fig. 6). These observations showed that the membrane rearrangements and structures associated with IBV infection in cultured cells were also present in cells associated with *ex vivo*-infected host tissues. This indicates that such membrane rearrangements and structures induced by IBV are not an artifact of cultured cells and play a role in the replication cycle of this virus in the infection of its natural host.

### Spherules are derived from zippered ER and have a channel from their interior to the cytoplasm.

**FIG 7:**
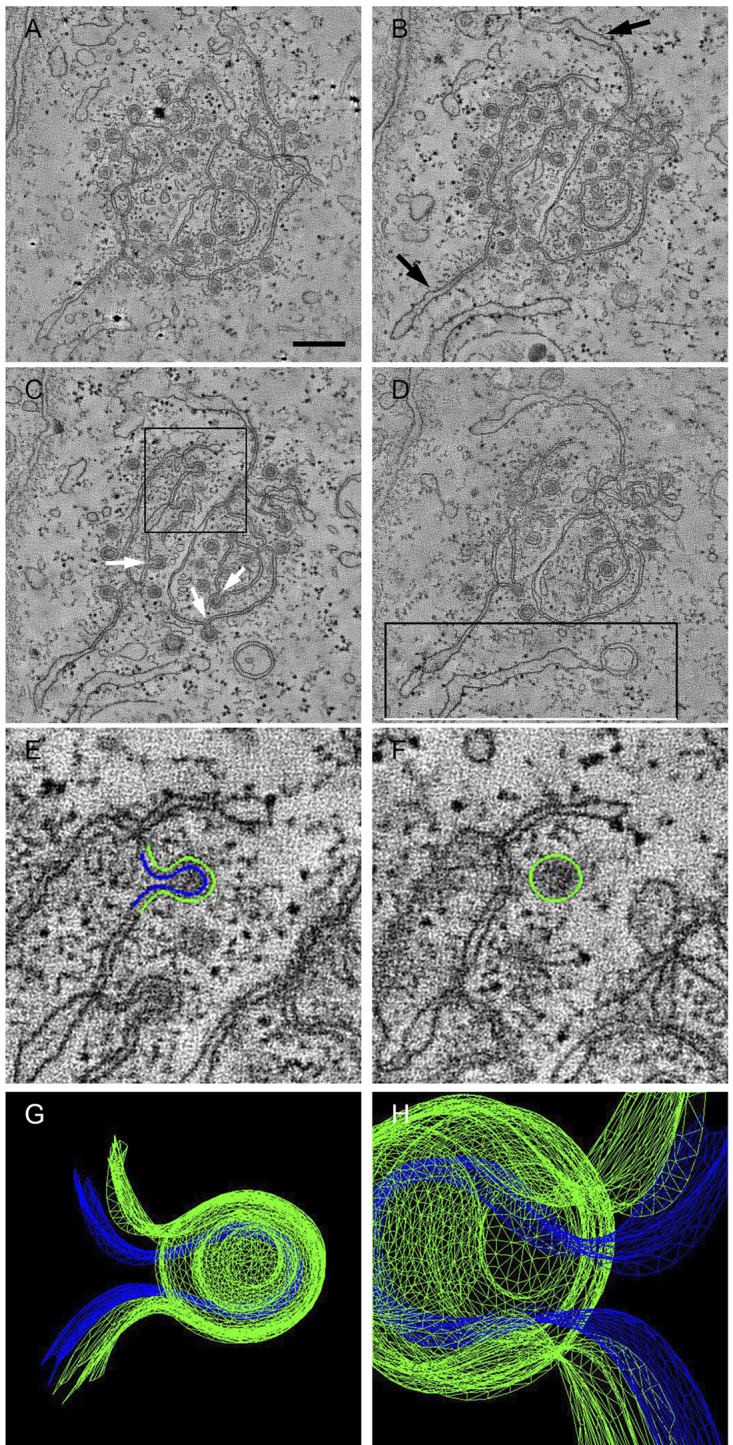
Spherules are derived from and connected to the zippered ER and have a channel connecting their interior to the cytoplasm. CK cells infected with IBV were chemically fixed at 16 hpi, and a dual-axis electron tomography data set was collected from a 300-nm-thick section. Images were collected at 1° intervals over a ±60° range using FEI T20 200 kV TEM and Acquire 3D software. Reconstruction and modeling were carried out using IMOD software. (A to D) Four images taken from the tomogram (for the full tomogram, see Movie S1 in the supplemental material). Collapsed ER membranes (arrows) (B) fold to form individual spherules with a clear channel connecting the cytoplasm and spherule interior (arrows indicate spherule necks) (C) and a connection between an ER tubule and a DMV (D). The area highlighted in panel C was modeled by tracing the two membranes in consecutive slices (outer membrane in green, inner membrane in blue). (E to F) Two images from the contour data set (for the full contour data set, see Movie S2). The three-dimensional mesh model of this spherule, represented by two images (G to H), clearly displays the open channel between the cell cytoplasm and the spherule center. For the model movie, see Movie S3. The scale bar in panel A indicates 50 nm.

The relationships between the different membrane structures observed during SARS-CoV or EAV infection have been evaluated using ET to generate a three-dimensional (3D) reconstruction (23, 28). These studies concluded that replication of both viruses was supported by a reticular network of rearranged membranes. We used ET to investigate in more detail the membrane rearrangements observed during IBV infection. No significant differences in the types of membrane structures induced by IBV in primary CK cells were observed by TEM at any time point after 7 hpi. Therefore, primary CK cells were infected and chemically fixed at 16 hpi, to increase the number of IBV-infected cells compared to earlier time points, and were assessed by ET. Analysis of these infected cells confirmed that spherules were comprised of a double membrane that was derived from and connected to the zippered ER. More importantly, ET identified that the novel spherule structures induced by IBV infection contained a 4.4-nm (±1.0 nm)-long channel or pore connecting their interior to the cytoplasm (Fig. 7; see also Movies S1 to S4 in the supplemental material), a feature not identified for any other coronavirus-induced structure. In addition, ET provided further evidence for the zippering together of the rough ER membranes to form zippered ER and that ribosomes were not associated with the zippered ER (Fig. 7 and Movies S1 to S4).

### IBV-induced DMVs exist as single vesicles or as networks connected to the ER via their outer membrane.

**FIG 8:**
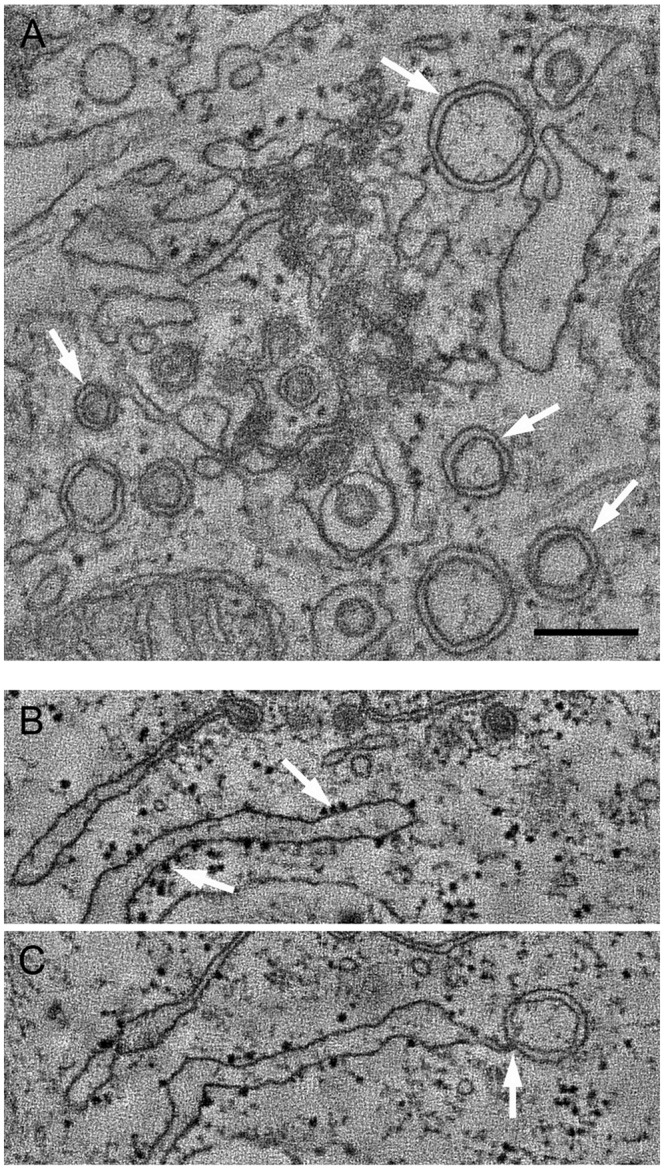
Double-membrane vesicles exist as single vesicles. (A) The tomographic reconstruction of an IBV-infected cell chemically fixed at 16 hpi shows an accumulation of individual DMVs which do not appear to be connected (arrows). For the full reconstruction, see Movie S4 in the supplemental material. The data set represented by panel A was collected under the same conditions as described for Fig. 7. (B and C) Double-membrane vesicles appear to be connected to rough ER. Ribosomes on the ER tubule are highlighted in panel B (arrows), and the connection between the ER tubule and DMV is demonstrated in panel C (arrow). Panels B and C represent still images taken from Movie S6, which in turn was extracted from the full tomogram represented by Movie S1, focusing on the area highlighted in Fig. 7D. The scale bar in panel A indicates 50 nm.

Analysis of IBV-infected CK cells using ET also demonstrated that for the majority of the IBV-induced DMVs, no links could be detected between the DMV membranes or with any other membrane and that DMVs instead appeared to exist as single vesicles (Fig. 8A; see also Movie S5 in the supplemental material). However, for a small proportion of DMVs, the outer membranes were observed to be connected to the ER (Fig. 8B and C and Movie S6), as observed for DMVs in SARS-CoV- and EAV-infected cells (23, 28). In all cases, no channels or pores were observed connecting the interior of the IBV-induced DMVs with the cytoplasm and, although ribosomes were detected on the membranes of the ER (Fig. 8B and Movies S5 and S6), no ribosomes were detected in association with the outer membrane of the DMVs (Fig. 8A and C).

## DISCUSSION

### IBV-induced zippered ER and spherules.

In this work, we have identified that novel zippered ER and associated 60- to 80-nm-diameter spherules are induced by IBV, structures that are not present in cells infected with betacoronaviruses. These structures were identified not only in a continuous mammalian cell line (Vero cells) but, more importantly, also in primary avian cells and in the epithelial cells of the chicken trachea using *ex vivo* organ cultures. Significantly, spherules were found to contain a 4.4-nm-long channel connecting their interior to the cytoplasm of the cell, a property not previously found in any coronavirus-induced membrane rearrangements. The channel diameter connecting IBV-induced spherules to the cytoplasm would be large enough to allow exchange of nucleotides and RNA products with the cytoplasm (14) and is consistent with the hypothesized requirements for a likely site of viral RNA synthesis. Spherules have been identified in a broad range of mammalian, plant, and insect cells infected with alphaviruses, bromoviruses, and nodaviruses. Cells infected with the alphaviruses SFV, Sindbis virus, and Western equine encephalomyelitis virus were observed to contain large cytoplasmic vacuoles lined with spherules on the inner surface and also at the plasma membrane (32–35). These alphavirus-induced spherules were comprised of a single membrane and were 50 nm in diameter. In addition, the spherules were shown to be the site of viral RNA synthesis (15, 34, 35) and are generated at the plasma membrane by the interaction of the viral nonstructural proteins and genomic viral RNA (15). In yeast cells infected with the bromovirus BMV, viral RNA synthesis was found to occur on ER membranes (36, 37). Expression of BMV nonstructural protein 1a alone or with nonstructural protein 2a and viral RNA resulted in the appearance of single-membrane spherules of 50 to 70 nm in diameter on the ER, and these were shown to be the site of viral RNA synthesis (38). Similarly, cells infected with the alphanodavirus FHV were observed to contain 50-nm-diameter single-membrane spherules on the outer mitochondrial membrane, with 10-nm-long channels connecting the content of the spherules to the cytoplasm (14, 39). The inner sides of the FHV-induced spherules were shown to contain viral RNA intermediates and multifunctional replication protein A and to be the site of RNA synthesis (14). Further work, studying the mechanism of formation of these spherules, showed that protein A and replication-competent RNA were required for induction of the spherules and that protein A must be functional for FHV RNA polymerase activity (40). Interestingly, when protein A was expressed alone, spherules did not form but, instead, the outer and inner mitochondrial membranes were zippered together, suggesting a possible intermediate step in spherule formation (40). These common features associated with viral replication complexes from other single-stranded positive-sense RNA viruses, including the zippering of cellular membranes and 50- to 80-nm-diameter spherules with a 4.4- to 10-nm-long channel connecting the inside of the spherule to the cytoplasm, may suggest that similar mechanisms have been adopted by IBV to generate sites for RNA synthesis.

Interestingly, only very minor percentages of viral polymerase, Nsp12, and IBV-induced dsRNA were observed to be colocalized at any time point postinfection. Consistent with its presence in the interior of DMVs, shielded from cellular detection (23, 41), IBV-induced dsRNA did not colocalize with ER or Golgi markers (data not shown). In addition, the pattern of IBV-induced dsRNA labeling altered during the course of the replication cycle, consistent with observations during infection of cells with SARS-CoV and MHV (23, 29), suggesting that IBV-induced dsRNA may also be located within the interior of DMVs, although direct evidence would be required to confirm this. Since Nsp12 must be present at the site of viral RNA synthesis, dsRNA possibly associated with DMVs is unlikely to be part of viral replication complexes in IBV-infected cells and, as a consequence, viral RNA synthesis may not be associated with DMVs but may instead occur on alternate membrane structures, possibly spherules.

### Other IBV-induced membrane rearrangements.

In addition to zippered ER and spherules, DMVs were also identified in IBV-infected cells. The IBV-induced DMVs closely resemble both in appearance and in size those identified in SARS-CoV-, MERS-CoV-, and MHV-infected cells (18–24). IBV belongs to a coronavirus genus different from that of these viruses, suggesting that the DMVs observed in coronavirus-infected cells are likely to be generated via an analogous mechanism. In addition, although the majority of DMVs were observed as single vesicles, some of the IBV-induced DMVs were shown to be linked to the ER via their outer membranes to form a network of membranes. The inner membranes of the DMVs observed in this study were found to be discrete structures with no apparent channel or pore connecting their core to the cytoplasm, consistent with observations for SARS-CoV-induced DMVs (23). As an effect of the fixation method used for some experiments, the electron-dense content of the IBV-induced DMVs was preserved and was shown to resemble the content of DMVs observed in cells infected with the arterivirus EAV fixed by the same method (28). This would further suggest that DMVs induced by viruses from the *Nidovirales* order are likely to be closely related structures and to perform similar functions in the life cycles of those viruses, potentially storing dsRNA (29). Interestingly, unlike the convoluted membranes observed during SARS-CoV infection, no links were observed between zippered ER and DMVs identified in IBV-infected cells, and although the two structures were often found in close proximity, they would appear to be discrete structures. No links were observed between the convoluted membranes and DMVs observed in MHV-infected cells (24), raising the possibility that a low frequency of connections, below levels of detection, may exist between convoluted membranes and DMVs observed in betacoronavirus-infected cells and also between zippered ER and DMVs in IBV-infected cells.

In IBV-infected cells, zippered ER with associated spherules and DMVs were observed both in clusters spread throughout the cytoplasm (Fig. 3A) and in perinuclear clusters (Fig. 5A). The size of clusters also ranged from small clusters of fewer than 10 spherules (Fig. 3A) to large regions containing up to 100 DMVs in addition to zippered ER and spherules (Fig. 5A). As membrane rearrangements were not detected until 7 hpi, it is not clear whether the size of the cluster or the location within the cell is dependent upon the stage of virus infection, as observed for the betacoronaviruses (23, 24). The total numbers of DMVs and of regions of zippered ER with spherules also differed greatly between cells, possibly due to the range of stages of virus infection present in cells at 16 or 24 hpi. In general, however, the total number of DMVs per cell section was observed to be far lower than that in SARS-CoV-infected cells (23) and is possibly more consistent with observations in MHV-infected cells (24).

Other membrane rearrangements that have been observed in coronavirus-infected cells include vesicle packets, tubular bodies, and cubic membrane structures (20, 23–25, 42). None of these were identified in IBV-infected cells, despite analysis at several time points postinfection. Tubular bodies were identified during MHV infection and were shown to contain the envelope (E) protein, but no other viral proteins, and so were suggested to be generated as a result of overexpression of E protein (24). Similarly, cubic membrane structures identified in MHV-infected cells were hypothesized to be generated by the overexpression and self-interaction of the spike (S) protein (24). It is possible that the E and S proteins are expressed to a lesser extent during IBV infection or that the effect of overexpression of these two IBV proteins does not result in membrane rearrangements in primary avian cells or Vero cells. The absence of these structures in IBV-infected cells, particularly convoluted membranes, highlights that, in contrast to previous suggestions (24), important differences exist in membrane rearrangements observed in cells infected with coronaviruses belonging to different genera. The effect must be virus driven, as different membrane rearrangements are observed in Vero cells when infected with SARS-CoV (23) versus MERS-CoV (18) or IBV.

The novel zippered ER observed during IBV infection resembles, to an extent, the paired membranes found in EAV-infected cells, which have also been shown to be derived from the ER. However, while DMVs in EAV-infected cells are smaller than those observed in coronavirus-infected cells and have been identified as being connected to the paired membranes (28), as are spherules to zippered ER in IBV-infected cells, they are distinct structures. The morphologies of DMVs in both EAV- and IBV-infected cells, despite differences in size, are very similar. Following the fixation of EAV- or IBV-infected cells by high-pressure freezing, both types of DMV were observed to contain a core of electron-dense material with a more electron-lucent halo between the core and the double membrane. Additionally, this electron-dense content was lost upon chemical fixation of both EAV- and IBV-infected cells. The spherules identified in IBV-infected cells, however, were observed to have a more evenly distributed content regardless of the fixation method used. In addition, and most importantly, the DMVs in both EAV- and IBV-infected cells have a discrete inner membrane, with no connections to the cytoplasm. In contrast, the 60- to 80-nm-diameter spherules identified in IBV-infected cells were found to have a 4.4-nm-diameter pore connecting their interior to the host cell cytoplasm, which clearly defines them as a distinct structure.

An interesting apparent difference between the membrane rearrangements and associated structures in cells infected with SARS-CoV, MHV, or IBV is seen in the timing of the onset of these rearrangements and structures. While the data generated for RNA synthesis, protein synthesis, and progeny release during IBV infection were broadly consistent with previous observations for MHV and SARS-CoV (24, 43–45), detection of membrane rearrangements in IBV-infected cells did not occur until 7 hpi with the sudden appearance of large regions within infected cells containing both DMVs and zippered ER with spherules. In contrast, membrane rearrangements were identified relatively early following infection with SARS-CoV or MHV in which DMVs were detectable from 2 hpi and convoluted membranes by 3 hpi (23, 24). We propose that early in IBV infection, replication occurs on small regions of zippered ER and associated spherules. These structures are initially very low in numbers and are therefore rare structures that are very hard to detect by electron microscopy. We hypothesize that when sufficient viral Nsps have been translated at later time points postinfection, a critical mass of viral-derived proteins is reached that concomitantly triggers an exponential increase in the number of replication structures, making them more easily identifiable. Such an event would result in the rapid appearance of rearranged membrane structures in IBV-infected cells such as was observed at 7 hpi. Indeed, the number of DMVs in both SARS-CoV- and MHV-infected cells was observed to dramatically increase during the course of infection (23, 24). Interestingly, RNA synthesis and turnover reaches a peak level at 7 hpi, consistent with the appearance of large areas of rearranged membranes within infected cells.

Overall, our results have highlighted important similarities between membrane structures induced by IBV and other related nidoviruses. However, important differences and novel membrane structures in IBV-infected cells compared to those induced by other coronaviruses and related nidoviruses have also been identified. These bear more similarity to membrane structures associated with confirmed RNA synthesis sites for other positive-sense single-stranded RNA viruses. Further work will be needed to analyze in detail whether spherules provide the site for IBV RNA synthesis as well as which viral proteins are responsible for membrane rearrangement.

## MATERIALS AND METHODS

### Cells, viruses, and antibodies.

Primary chick kidney (CK) cells were produced from 2-to-3-week-old specific-pathogen-free (SPF) Rhode Island Red chickens (46). Tracheal organ cultures (TOCs) were produced from 19-day-old SPF embryos (47, 48). The apathogenic molecular clone of IBV, Beau-R, has been described previously (49). BeauR-hRluc-Δ5a was generated using the IBV reverse genetics system and comprises a Beau-R backbone in which accessory gene 5a has been deleted and replaced with the Renilla luciferase gene (27). The monoclonal J2 anti-dsRNA antibody was purchased from English and Scientific Consulting Bt. Anti-nsp12 was generated by immunizing 2 rabbits with a peptide corresponding to amino acids 925 to 940 (EQEFYENMYRAPTTLQ) of a viral RNA-dependent RNA polymerase, Nsp12, from IBV Beau-R. Antisera were pooled from the two rabbits and were affinity purified against the immunogen. Goat anti-rabbit IgG Alexa Fluor 488 and goat anti-mouse IgG Alexa Fluor 568 secondary antibodies were purchased from Invitrogen.

### Immunofluorescence labeling.

CK cells were seeded onto glass coverslips and infected with the IBV Beau-R strain at a high MOI (greater than 40) to maximize the number of infected cells. At various time points postinfection, the cells were fixed in 4% paraformaldehyde–phosphate-buffered saline (PBS) for 20 min at room temperature. The fixed cells were permeabilized in 0.1% Triton X-100–PBS for 15 min and then incubated in 0.5% bovine serum albumin (BSA)–PBS blocking solution for 1 h. The cells were then incubated in primary antibody diluted in blocking solution for 1 h, washed three times with PBS, incubated in secondary antibody diluted in blocking solution for 1 h, and washed a further three times in PBS. Finally, nuclei were stained using 4',6-diamidino-2-phenylindole (DAPI) diluted in water and coverslips were mounted onto glass slides. Primary antibodies were diluted as follows: anti-dsRNA antibody was diluted 1:1,000, and anti-nsp12 was diluted 1:1,500. Secondary antibodies were diluted 1:200. Slides were imaged using a Leica SP5 confocal laser scanning microscope (CLSM) with a DM6000 microscope stand using LAS AF software (version 2.6.0.7266; Leica Microsystems CMS GmbH). For colocalization analysis, z-stacks covering whole cells at 0.29-µm steps were imaged. Analysis was performed on five fields of view using the polygon model of the Coloc module of Imaris 7.6 software (Bitplane Scientific Software). Images presented represent single-slice images and not z-stacks.

### RNA isolation and 2-step qRT-PCR.

CK cells were infected with IBV Beau-R at a MOI of 10. After 24 h, cells were harvested and lyzed using a QIAgen Tissuelyser II system. RNA was extracted using RNeasy columns (QIAgen), following the manufacturer’s instructions and including on-column DNAse treatment using an RNase-free DNase set (QIAgen). Quantitative RT-PCR reactions were performed to quantify levels of genomic 5′ UTR (50) and the N protein sgRNAs, which include both positive- and negative-sense RNAs. Total RNA was quantified, and 300 ng of RNA was used for each reverse transcription reaction. Standard curves were performed to allow absolute quantitation of IBV RNA copy numbers based on cDNA levels using plasmids containing the sequence amplified by each set of primers. Reverse-transcription reactions were performed using Taqman reverse transcription reagents (Applied Biosystems), following the manufacturer’s instructions and using primers specific for IBV 5′ UTR or IBV N sgRNA. Quantitative PCR was performed using Taqman Fast Universal PCR 2× Master Mix (Applied Biosystems) and including 125 nM final probe and 500 nM final primers. Primer and probe sequences to detect 5′ UTR were as follows: IBV5′GU391 (forward), 5′-GCTTTTGAGCCTAGCGTT-3′; IBV5′GL533 (reverse), 5′-GCCATGTTGTCACTGTCTATTG-3′; and IBV 5′ G probe, 5′-6-carboxyfluorescein (FAM)-CACCACCAGAACCTGTCACCTC-6-carboxytetramethylrhodamine (TAMRA)-3′. Primer and probe sequences to detect N sgRNA were as follows: Leader F, 5′ CTAGCCTTGCGCTAGATTTTTAACT 3′; N sgRNA R, 5′ GAGAGGTACACGCGGGACAA 3′; and N sgRNA probe, 5′-FAM-ACAAAGCAGGACAAGCA-MGB-NFQ-3′.

### Luciferase activity assay.

CK cells were infected at a high MOI (approximately 35) with BeauR-hRluc-Δ5a. After a 1-h incubation, cells were washed four times using PBS and incubated in 1× BES media [1× modified Eagle's medium (E-MEM, Sigma), 0.3% tryptose phosphate broth (TPB, BDH), 0.2% bovine serum albumin (BSA, Sigma), 20 mM BES (*N,N*-bis(2-hydroxyethyl)-2-aminoethanesulphonic acid, Sigma), 0.21% sodium bicarbonate, 2 mM l-glutamine, 250 U/ml nystatin, and 100 U/ml penicillin and streptomycin]. Cell lysates were harvested at hourly intervals from 2 hpi until 8 hpi following the Renilla luciferase assay system (Promega). Renilla luciferase assay was performed using a Glomax 20/20 luminometer (Promega) following the manufacturer’s instructions.

### Viral growth curve.

CK cells were infected with the IBV Beau-R strain at a MOI of 10 and incubated at 37°C for 1 h. Cells were washed four times using PBS to completely remove the inoculum and were incubated in 1× BES media. Cell supernatants were harvested at hourly intervals from 2 hpi until 8 hpi, and the presence of progeny virus was assayed by a plaque assay on CK cells.

### Transmission electron microscopy of chemically fixed cells.

Infected Vero or CK cells on Thermanox coverslips (VWR International, United Kingdom) or whole TOCs were fixed for 60 min in phosphate-buffered 2% glutaraldehyde solution before being fixed for a further 60 min in aqueous 2% osmium tetroxide at room temperature. Following a dehydration series at room temperature in ethanol (70% for 30 min, 90% for 15 min, and 100% three times for 10 min each wash), coverslips were washed with propylene oxide for 10 min before being infiltrated for 60 min with 50% epoxy resin (Agar Scientific, United Kingdom) in propylene oxide. After a further 60 min in 100% epoxy resin, coverslips were placed (cell side up) in 18-mm-diameter plastic cups (Taab, United Kingdom), covered in fresh epoxy resin, and polymerized at 60° C for 18 h. After removal of the plastic cups, the coverslips were peeled off while the resin was still warm, leaving the cells inside the resin block. The blocks were trimmed and sectioned en face so that all cells in any particular section were cut at similar orientations and depths (Z dimension). Sections (60 μm) were collected midheight (Z dimension) through the cells (too low, and cells were seen to contain mostly actin bundles; too high, and cells were seen to contain mostly nucleus surrounded by a small area of cytoplasm). Sections were cut and grid stained with uranyl acetate and lead citrate using EMStain AC20 (Leica Microsystems) before being imaged at 100 kV in an FEI Tecnai 12 TEM with a TVIPS F214 digital camera.

### High-pressure freezing and freeze-substitution.

CK cells were seeded onto 3-mm- or 6-mm-diameter sapphire glass discs (Leica Microsystems, United Kingdom) which had been incubated with fetal calf serum at 37°C for 60 min and were infected for the required length of time. Discs were then frozen in Bal-tec HPM010 HPF or Leica HPM100 HPF and stored in liquid nitrogen prior to freeze-substitution as described previously (51). Briefly, the sapphire discs were allowed to warm to −90°C, at which time the cold freeze-substitution medium of 2% uranyl acetate in acetone was added for 60 min. The temperature was allowed to rise to −50° C over a period of 2 h for infiltration of Lowicryl HM20 resin (Agar Scientific). Cells on discs were taken through a graded series of resin concentrations starting with 25% resin in acetone (30 min) followed by 50% (30 min) and 75% (30 min) and three times in 100% (30 min each) resin washes. All steps were carried out using a Leica AFS unit. Discs were mounted cell side up in flat embedding molds, covered in acrylic resin, sealed with a UV-transparent plastic cover to eliminate oxygen, and exposed to UV light for 48 h at −50°C followed by another 48 h at room temperature. After polymerization, sapphire discs were removed from the solid resin blocks by immersing them briefly in liquid nitrogen. Thin sections of the resulting resin blocks (60 nm) were cut en face using Leica Microsystems Ultracut E and imaged without contrasting in an FEI Tecnai 12 TEM at 100 kV using a TVIPS F214 digital camera.

### Electron tomography.

Three-hundred-nanometer sections of infected CK cells prepared by the chemical fixation protocol described above were cut and collected on copper single-slot grids coated with Pioloform film. Unstained sections were exposed to a solution of 15-nm-diameter gold fiducial markers for 5 min before being dried and loaded into the Fischione double-tilt holder. Dual-axis datasets were collected using an FEI T20 TEM at 200 kV and an Eagle 4 k camera. Images were collected at 1° increments over a range of ±60° before the sample was rotated through 90°, and another data set was under collected the same conditions, creating 242 image datasets. The FEI data collection module of the Inspect3D software package was used to automatically collect each series. Images within each series were aligned and used to create a tomogram 3D distribution of stain density. The two tomograms were then combined to produce a single, dual-axis reconstruction of the region of interest. Image alignment, tomogram production, and tomogram combination were carried out using the IMOD software package (52). The 3D reconstructions were segmented using IMOD, and membranes were traced by hand on consecutive Z slices within a tomogram using a Wacom Cintiq 21 UX interactive graphics tablet and pen. Measurements of the spherule pore size were taken from the central Z slice of each spherule using IMOD software.

## SUPPLEMENTAL MATERIAL

Movie S1 Tomographic reconstruction of part of an IBV-infected CK cell chemically fixed after 16 hpi. The reconstruction shows an area of zippered ER membranes and spherules. Download Movie S1, MOV file, 8.9 MB

Movie S2 Movie of part of the reconstruction in Movie S1, highlighting a single spherule. The tomogram clearly shows the 2 zippered ER membranes pinching out to form a spherule with a channel into the interior. Download Movie S2, MOV file, 6 MB

Movie S3 A small area of Movie S1 was modeled by tracing the spherule membranes in consecutive sections using IMOD software and a Wacom graphics tablet and pen. This video combines part of the reconstruction with the contours. Download Movie S3, MOV file, 6 MB

Movie S4 Movie showing the three-dimensional model created from the contour map in Movie S3. The outer face of the spherule is represented in green and the inner face in blue. The channel linking the inside of the spherule with the cell cytoplasm is clear. Download Movie S4, MOV file, 9.8 MB

Movie S5 Electron tomographic reconstruction of part of an IBV-infected CK cell. The area displayed in the reconstruction contains several double-membrane vesicles; however, they do not appear to be linked. Download Movie S5, MOV file, 15 MB

Movie S6 Movie of part of the reconstruction of Movie S1 highlighted in Fig. 8C. The tomogram clearly shows part of the endoplasmic reticulum connecting with the outer membrane of a double-membrane vesicle. Download Movie S6, MOV file, 11.9 MB
